# Nursing care for adult patients with chest drainage: a scoping review*

**DOI:** 10.1590/1980-220X-REEUSP-2024-0017en

**Published:** 2024-09-30

**Authors:** Elisiane Goveia da Silva, Bárbara Rodrigues Araujo, Raphaela de Matos Borges, Tainara Wink Vieira, Cristiane Cardoso de Paula, Rita Catalina Aquino Caregnato

**Affiliations:** 1 Universidade Federal de Ciências da Saúde de Porto Alegre, Departamento de Enfermagem, Porto Alegre, RS, Brazil.; 2 Hospital Nossa Senhora da Conceição, Porto Alegre, RS, Brazil.; 3 Universidade Federal de Santa Maria, Departamento de Enfermagem, Santa Maria, RS, Brazil.

**Keywords:** Nursing Care, Chest Tubes, Intensive Care Units, Atención de Enfermería, Tubos Torácicos, Unidades de Cuidados Intensivos

## Abstract

**Objective:**

To map the nursing care recommended for adult patients undergoing chest drainage.

**Method:**

Scoping review. Included studies in Portuguese, English and Spanish, with no time frame, which answered the research question: what nursing care is indicated for adult patients with chest drainage admitted to intensive care? Selection in the MEDLINE/PubMed, Embase/Elsevier, Web of Science/Clarivate, Scopus/Elsevier, CINAHL/Ebsco and LILACS/BVS databases. No data from gray literature was included.

**Results:**

Of the 973 articles identified, 21 were selected. The most frequently cited precautions included: filling the collection bottle with distilled water or saline solution, leaving the distal end of the stem submerged 1.5 to 2.5 centimeters; monitoring vital signs; pain management; and proper positioning of the system. There were differences in the indication for clamping the system and milking the drain/drainage system.

**Conclusion:**

60 nursing tasks were mapped, 13 of which were carried out prior to inserting the drain, nine during insertion and 38 after insertion of the chest drain.

## RESUMEN

**Objetivo:**

Mapear los cuidados de enfermería recomendados para pacientes adultos sometidos a drenaje torácico.

**Método:**

Revisión de alcance. Se incluyeron estudios en portugués, inglés y español, sin marco temporal, que respondieran a la pregunta de investigación: ¿qué cuidados de enfermería se recomiendan para pacientes adultos con drenaje torácico ingresados en cuidados intensivos? Selección en las bases de datos MEDLINE/PubMed, Embase/Elsevier, Web of Science/Clarivate, Scopus/Elsevier, CINAHL/Ebsco y LILACS/BVS. No se incluyeron datos de literatura gris.

**Resultados:**

De los 973 artículos identificados, se seleccionaron 21. Las precauciones citadas con más frecuencia incluían: llenar el frasco de recogida con agua destilada o solución fisiológica, dejando el extremo distal del vástago sumergido entre 1,5 y 2,5 centímetros; monitorización de las constantes vitales; tratamiento del dolor; y colocación adecuada del sistema. Hubo diferencias en la indicación de pinzar el sistema y ordeñar el sistema de drenaje/drenaje.

**Conclusión:**

Se mapearon 60 tareas de enfermería, 13 de las cuales se realizaron antes de la inserción del drenaje, nueve durante la inserción y 38 después de la inserción del drenaje torácico.

## INTRODUCTION

Evidence-Based Practice (EBP) is aimed towards improving clinical effectiveness and supporting health professionals in decision-making, adopting three main elements in its approach: scientific evidence, clinical experience and patient preferences^(1,2)^.

The nurses’ role in the development, application and evaluation of health technologies has evolved in the production of protocols and educational materials based on EBP. Ordinance No. 2.510/GM of 2005 defines health technologies as: medicines, materials, equipment and procedures, organizational, educational, information and support systems, programs and care protocols, through which health care is provided to the population^(3)^.

The technologies used by the nursing team to support daily practices are classified as products and/or processes. Products are defined as computerization, information and artifacts; and processes correspond to structured knowledge, such as theories and educational tools. In this way, care protocols are considered health technologies and are indicated in the organization of nursing processes and the provision of appropriate care in a safe and efficient manner^(4)^.

Considered a health technology, thoracic drainage (TD) consists of installing a tubular drain in the pleural cavity connected to a water seal system, with the aim of draining the anomalous contents of the pleural space, in order to re-expand the lungs. If necessary, a second bottle can be attached to the system and connected to a continuous suction network, with the aim of controlled and continuous suctioning, helping to maintain the balance of negative intrathoracic pressure^(5,6)^. TD is considered a safe and effective technology, widely used in elective and/or emergency procedures to treat pulmonary complications such as pneumothorax, hemothorax, complicated pleural effusion, empyema, chylothorax^(7)^ and in the postoperative period of thoracic and mediastinal surgeries^(6)^.

Despite being a common technology in the hospital setting, TD is not without its complications. A descriptive study carried out in Brazil with the aim of identifying the predictors of TD complications in trauma patients found a complication rate of 26.3%^(8)^. The main complications associated with the procedure include: poor positioning of the drain, requiring a new procedure; residual hemothorax/pneumothorax; pneumonia and infection; and the orifice remaining outside the chest cavity^(8)^.

Nursing is involved in all stages of patient care, and it is essential to implement evidence-based care that promotes patient safety, with a view to effective treatment and the prevention of complications^(9)^.

Researchers at the Nigerian Semi-urban University Hospital, who sought to assess the level of knowledge about chest tube care among nurses, covering everything from anatomical aspects to post-procedure care, found that nursing professionals believe in the importance of training in chest tube care, but the majority (66.7%) had not received refresher training on the subject^(10)^. In addition, around 45% of nurses did not know or were not sure that the level of floating fluid in the drainage tube was indicative of the device working properly^(10)^. It is therefore essential to produce scientific evidence that can support the construction of nursing care protocols for patients using TDs, promoting the qualification of care and making care safer.

Faced with this problem, this study aimed to map out the nursing care applied to adult patients undergoing TD.

## METHOD

### Type of Study

This is a scoping review, following the recommendations of the Joanna Briggs Institute^(11,12)^, a course of action indicated for mapping out concepts and presenting a broad view of the evidence pertaining to a given topic, a strategy that demonstrated affinity with the objective of the study^(11,12)^. The protocol for this review was registered on the Open Science Framework (OSF) platform under DOI: 10.17605/osf.io/T8RW9 and previously published^(13)^. The following review question was defined: “What nursing care is indicated for adult patients with TD admitted to intensive care?”.

In order to guarantee the quality and transparency of the writing, we used the Preferred Reporting Items for Systematic Reviews and Meta-Analyses Extension for Scoping Reviews (PRISMA-ScR) Checklist^(14,15)^.

### Selection Criteria

The review included publications with no time frame, quantitative and qualitative studies, review articles, clinical guidelines and therapeutic protocols that meet the acronym PCC, where: P (population) adult patients with chest drainage; C (concept) nursing care; and C (context) intensive care.

Editorials, letters to the editor, opinion articles and narrative reviews were excluded because, although they play a fundamental role in continuing education, they do not have a methodology that allows data to be reproduced, nor do they provide answers to more specific research questions^(16)^. Other sources of gray literature were not evaluated, as it was considered that the recurrence of data would indicate information saturation^(17)^.

### Data Collection

The search strategies were developed by the researchers and reviewed by a librarian. Initially, a search strategy was developed for MEDLINE/PubMed and adaptations were made for the other databases, namely: Embase/Elsevier, Web of Science/Clarivate, Scopus/Elsevier, Cumulative Index to Nursing and Allied Health Literature (CINAHL)/Ebsco and Latin American and Caribbean Health Sciences Literature (LILACS)/Virtual Health Library (VHL). The search terms were defined using the Descriptors in Health Sciences (DeCS)/Medical Subject Headings (MeSH) and non-controlled descriptors, combined using the Boolean operators AND and OR (Chart 1).

**Chart 1:** Search terms used in the MEDLINE/PubMed database – Porto Alegre, RS, Brazil. 2023.

Database	Strategy
**MEDLINE/PubMed**	(Nursing Care[mh:noexp] OR Nursing[mh] OR Nursing, Team[mh] OR Nursing Staff, Hospital[mh] OR Nursing Service, Hospital[mh] OR Nursing Assessment[mh] OR Nursing Process[mh:noexp] OR Nurses[mh] OR Nursing[sh] OR Nursing*[tiab] OR Nurse*[tiab]) *AND* (Thoracostomy[mh] OR Thoracostom*[tiab] OR Thoracic drain*[tiab] OR Thorax drain*[tiab] OR Chest drain*[tiab] OR Pleural drain*[tiab] OR ((Chest Tubes[mh] OR Chest Tube*[tiab]) AND (Drainage[mh] OR Drain*[tiab] OR Suction[mh] OR Suction*[tiab])))
**Embase/Elsevier**	(‘nursing care’/exp OR ‘nursing’/exp OR ‘nursing staff’/exp OR ‘nurse’/exp OR Nursing*:ti,ab,kw OR Nurse*:ti,ab,kw) *AND* (‘thoracostomy’/exp OR ‘thorax drainage’/exp OR Thoracostom*:ti,ab,kw OR ‘Thoracic drain*’:ti,ab,kw OR ‘Thorax drain*’:ti,ab,kw OR ‘Chest drain*’:ti,ab,kw OR ‘Pleural drain*’:ti,ab,kw OR ((‘chest tube’/exp OR ‘Chest Tube*’:ti,ab,kw) AND (‘suction drain’/exp OR ‘drain’/de OR ‘suction’/de OR ‘suction drainage’/de OR Drain*:ti,ab,kw OR Suction*:ti,ab,kw))) *AND* *([embase]/lim NOT ([embase]/lim AND [medline]/lim))*
**Web of Science**	TS=(Nursing* OR Nurse*) *AND* TS=(Thoracostom* OR “Thoracic drain*” OR “Thorax drain*” OR “Chest drain*” OR “Pleural drain*”) OR (TS=”Chest Tube*” AND TS=(Drain* OR Suction*))
**Scopus**	TITLE-ABS-KEY(Nursing* OR Nurse*) *AND* (TITLE-ABS-KEY(Thoracostom* OR “Thoracic drain*” OR “Thorax drain*” OR “Chest drain*” OR “Pleural drain*”) OR (TITLE-ABS-KEY(“Chest Tube*”) AND TITLE-ABS-KEY(Drain* OR Suction*)))
**CINAHL**	(MH (“Nursing Care” OR Nursing OR “Nursing, Team” OR “Nursing Staff, Hospital” OR “Nursing Service, Hospital” OR “Nursing Assessment” OR “Nursing Process” OR “Nurses”) OR TI (Nursing* OR Nurse*) OR AB (Nursing* OR Nurse*) OR SU (Nursing* OR Nurse*)) *AND* (((MH Chest Tubes OR TI “Chest Tube*” OR AB “Chest Tube*” OR SU “Chest Tube*”) AND (MH (Drainage OR Suction) OR TI (Drain* OR Suction*) OR AB (Drain* OR Suction*) OR SU (Drain* OR Suction*))) OR MH thoracostomy OR TI (Thoracostom* OR “Thoracic drain*” OR “Thorax drain*” OR “Chest drain*” OR “Pleural drain*”) OR AB (Thoracostom* OR “Thoracic drain*” OR “Thorax drain*” OR “Chest drain*” OR “Pleural drain*”) OR SU (Thoracostom* OR “Thoracic drain*” OR “Thorax drain*” OR “Chest drain*” OR “Pleural drain*”)))
**BVS**	(mh:(“Nursing Care” OR Nursing OR “Nursing, Team” OR “Nursing Staff, Hospital” OR “Nursing Service, Hospital” OR “Nursing Assessment” OR “Nursing Process” OR N02.360.650*) OR ti:(Enfermage* OR Nursing* OR Enfermeria* OR Enfermera* OR Enfermero* OR Enfermeir*) OR ab:(Enfermage* OR Nursing* OR Enfermeria* OR Enfermera* OR Enfermero* OR Enfermeir*)) *AND* (mh:(“Chest Tubes” OR Thoracostomy) OR ti:(“Chest Tubes” OR “Chest Tube” OR “Tubos toracicos” OR “Tubo toracico” OR Thoracostom* OR Toracostom*) OR ab:(Thoracostom* OR Toracostom*) OR (tw:(Thorax* OR Thoracic* OR Torax OR Toracic* OR Pleura* OR Chest*) AND (tw:Drenage* OR Drenaje* OR Dreno* OR Drain* OR Suction* OR Succao OR Succoes OR Succion*)))

The studies were imported on January 29, 2023, in files using reference manager format (RIS or NBIB), into the Covidence^®^ software, a tool that helps to stratify the items according to each selection stage, meeting the methodological recommendations of this study. The studies were blindly selected by two independent reviewers with experience in the subject. Disagreements were resolved by discussion between the reviewers, and in cases where no consensus was reached, a third reviewer was consulted.

At first, articles identified as duplicates were excluded. The titles, abstracts and full articles were then read, respectively. In addition, on October 19, 2023, the reference lists of the selected studies were checked in order to identify potential publications that were not identified by the initial search strategy adopted and/or publications that may have occurred after the initial date of importing the studies^(11)^.

### Data analysis and Processing

To extract the data, Microsoft Excel^®^ software was used to characterize the studies in the final sample, with the following information: study title, year of publication, country of origin, objective, design and population. The recommendations were extracted into three temporal sub-themes: before, during and after chest tube insertion. The studies were identified using the sequential code E1 to E26 and, after sequential coding, the additional ones with the acronym “AD”.

Scoping reviews are generally conducted to provide an overview of existing evidence, regardless of methodological quality or risk of bias^(11)^. The analysis of the data extracted was descriptive in nature. The characterization data of the included studies was analyzed using descriptive statistics. These recommendations were then described in detail^(18)^. The results are presented in tables and narrative summaries. The discussion section synthesizes the evidence found during the review in order to explore it and compare it to the existing literature^(19)^.

### Ethical Aspects

All the fundamental ethical and scientific requirements for carrying out the study were respected, based on the guidelines and provisions contained in Law No. 12,853, of August 14, 2013, which provides for the collective management of copyright and makes other provisions, ensuring the authorship of the studies used, as well as the authenticity of the authors’ ideas, concepts and definitions, in order to preserve copyright^(20)^.

## RESULTS

**Figure 1:**
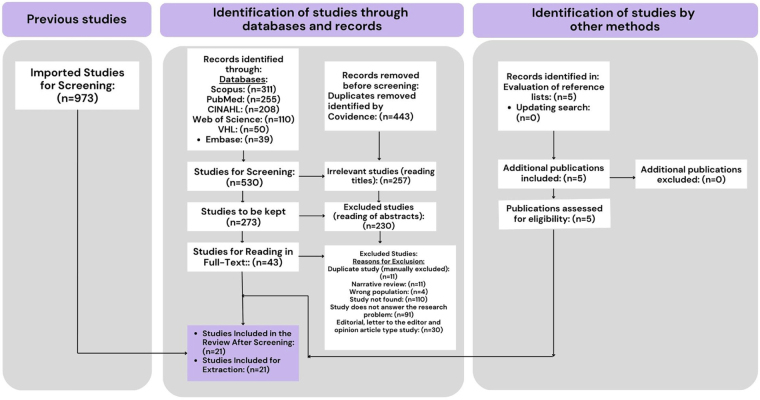
Flowchart of study selection – Porto Alegre, RS, Brazil, 2023^(15)^.

The selection process began with 973 studies mapped in the databases, from which 21 studies were selected for the extraction of recommendations. The studies included were in Portuguese, English and Spanish, and five were added by manual search, through evaluation of the reference lists, because they answered the review question (Figure 1). It should be noted that among the excluded studies, there were a total of 110 studies whose full texts could not be found for evaluation, even after attempting to contact the authors.

Of the studies selected, eight were literature reviews, eight observational cross-sectional studies, two systematic reviews, two randomized studies and one content validation study. In terms of origin, seven were produced in South America, three in the United States, three in Europe, three in the Middle East, two in Egypt, two in Asia and one in Australia (Chart 2).

**Chart 2:** Characterization of the selected studies – Porto Alegre, RS, Brazil. 2023.

Code	Study title	Year	Country of origin	Study objective	Design	Population
E1	Nursing management of chest drains: a systematic review ^(21)^	2001	Australia	To present the best available research evidence on the nursing management of chest drains.	Systematic review	Eight randomized controlled trials.
E2	Chest drain removal in the postoperative period of cardiac surgery^(22)^	2005	Brazil	To draw up a roadmap for the removal of chest drains by nurses in coronary and cardiac surgery units.	Literature review	There is no total number of studies included.
E3	To strip or not to strip? Physiological effects of chest tube manipulation^(23)^	2007	United States of America	Summarize the scientific evidence on chest tube milking.	Literature review	Five original research studies, one systematic review, two exploratory descriptive studies.
E4	Nursing management of patients with a chest drain^(24)^	2008	United Kingdom	To examine the role of nursing in the management of chest drainage, from insertion to removal, and to include aspects of pain control characteristic of a functional chest drain.	Literature review	Between 2000-2007, 15 articles; however, the authors increased the period to 1995-2007 and did not inform the total number of articles.
E5	The influence of the use of clamps on the accumulation of clots in tubular pleural drains^(25)^	2008	Brazil	To assess the influence of the use of a cuff on the accumulation of clots inside pleural drains.	Prospective field study	53 patients (53 pleural tubes).
E6	The Effect of Cold Application in Combination with Standard Analgesic Administration on Pain and Anxiety during Chest Tube Removal: A Single-Blinded, Randomized, Double-Controlled Study^(26)^	2010	Türkiye	To investigate the effect of cold application on pain and anxiety during chest tube removal in patients undergoing cardiac surgery.	Single-blind randomized study	90 patients admitted to the cardiovascular and thoracic surgical ICU.
E7	*Assistência de enfermagem na drenagem torácica: revisão de literature* (Nursing care in chest drainage: a literature review)^(27)^	2011	Brazil	A literature review on nursing care in chest drainage.	Literature review	No total number of studies included.
E8	Analytical Study on Practices Related to Care of Water Sealed Chest Drainage System^(28)^	2012	India	To analyze the prevalent practices in the care of the water-sealed thoracic drainage system according to the opinions of experts and against the available evidence.	Observational study	14 specialists.
E9	Chest tube care in critically ill patient: A comprehensive review^(29)^	2015	Egypt	Know the risks associated with chest tube insertion. Describe how to prepare the chest drainage unit, carry out continuous patient assessments, properly document and solve possible problems related to the use of chest drains.	Literature review	There is no total number of studies included.
E10AD	*Manipulação de drenos mediastinais e pleurais: existe evidência científica?* (Manipulation of mediastinal and pleural drains: is there scientific evidence?)^(30)^	2015	Brazil	To verify the existence of scientific evidence on the manipulation of pleural and mediastinal drains in cardiac surgery.	Systematic review	Six articles, including a review, a controlled clinical trial, a controlled experimental study and two descriptive studies.
E11	Institutional protocol to standardize the chest drainage system management, from surgery to nursing care, at a regional hospital in northern Paraná^(31)^	2016	Brazil	To evaluate the management of closed chest drainage systems by analyzing adult patients at the Regional University Hospital of Maringá, Paraná, to standardize the chest drainage care protocol and minimize complications.	Prospective observational study	We observed 100 chest drains from 83 patients.
E12AD	Nurses’ knowledge and Practice regard Care of Patient with Chest Drains in Sudan Heart Center, Khartoum, Sudan^(32)^	2016	Sudan	To evaluate nurses’ knowledge and practice in relation to patients connected to chest drainage.	Quantitative descriptive study	50 nurses.
E13	Variation in nurse self-reported practice of managing chest tubes: A cross-sectional study^(33)^	2018	China	To reveal the self-reported practice of managing chest drains by nurses and to define the decision makers for these practices.	Cross-sectional study	296 nurses.
E14	*Intervenção de enfermagem: cuidados com dreno torácico em adultos no pós-operatório* (Nursing intervention: chest tube care in post-operative adults)^(34)^	2018	Brazil	Validate the nursing activities for the intervention established by the Nursing Interventions Classification “chest tube care”.	Content validation study	30 nurses specialists.
E15	Comparative Evaluation of Chest Tube Insertion Site Dressings: A Randomized Controlled Trial^(35)^	2019	United States of America	To compare various types of dressings and procedures after the placement of thoracic and mediastinal drains.	Prospective randomized study	127 patients from three Intensive Care Units and 236 chest drains. Three groups were sampled, each with a different coverage and change frequency.
E16	Evidence-based management of patients with chest tube drainage system to reduce complications in cardiothoracic vascular surgery wards^(36)^	2021	United States of America	Implement best practices to provide safe and effective care for patients with chest drainage systems in cardiothoracic wards.	Literature review	No number of studies included.
E17	Interdisciplinary teamwork for chest tube insertion and management: an integrative review^(37)^	2021	France	To identify articles related to the interdisciplinary management of thoracotomy.	Integrative review	19 articles.
E18AD	*Boas práticas de enfermagem na utilização de dreno de tórax: revisão integrativa* (Good nursing practices in the use of chest drains: an integrative review)^(10)^	2021	Brazil	To identify in the scientific literature the best nursing practices related to the use of chest drains in adult intensive care units.	Integrative review	Five articles, one quantitative and four quantitative.
E19AD	Chest drains: prevalence of insertion and ICU nurses’ knowledge of care^(38)^	2021	Jordan	To describe the prevalence rate of chest tube insertion in Jordanian ICUs and to assess the level of knowledge of Jordanian nurses about chest tube care.	Descriptive non-experimental cross-sectional study	1973 files analyzed and questionnaire with 225 nurses.
E20AD	Nurses’ Knowledge Levels About the Care of the Patients with Chest Tube^(39)^	2021	Türkiye	To determine nurses’ level of knowledge about caring for patients with chest drains.	Descriptive study	152 nurses.
E21	Development and validation of the first performance assessment scale for interdisciplinary chest tube insertion: a prospective multicenter study^(40)^	2022	Germany	To validate an interdisciplinary performance assessment scale for chest tube insertion developed from an analysis of the literature.	Multicenter prospective study	40 videos of simulation sessions carried out by 80 participants.

A total of 60 recommendations were extracted, divided into 13 before, nine during and 38 after insertion of the TD. All the recommendations were organized according to the respective studies, the source of the information and the total number of studies covering each item (Chart 3).

**Chart 3:** Recommendations extracted from the final sample – Porto Alegre, RS, Brazil, 2023.

N°	Pre-insertion TD recommendations	Code of studies
**1**	Double-check the patient’s identification.	E21^(40)^
**2**	Inform the patient about the procedure and how they can help.	E7^(27)^, E17^(37)^, E21^(40)^
**3**	Inform the family about appropriate care.	E14^(34)^
**4**	Apply informed consent form.	E7^(27)^, E17^(37)^, E21^(40)^
**5**	Check for allergies.	E7^(27)^, E21^(40)^
**6**	Prepare the appropriate materials for inserting the TD: antiseptic solution (we suggest using alcoholic chlorhexidine solution because it is more cost-effective), sterile apron, mask and gloves, sterile drapes, sterile chest tube tray, local anesthetic, needles, syringes, scalpel with blade, curved kelly clamps, chest drain, drainage system, sharps waste, dressing material.	E17^(37)^, E21^(40)^
**7**	Simple hand hygiene with antiseptic.	E14^(34)^, E21^(40)^, E18AD^(10)^
**8**	Position the patient comfortably in bed: with the arm on the side where the drain will be inserted, placed behind the head so as to expose the armpit. The surgeon may ask for a pillow or blanket folded under the patient’s shoulder blades to elevate the chest and facilitate access.	E4^(24)^, E7^(27)^, E9^(29)^, E21^(40)^
**9**	Fill the collection bottle with distilled water or saline solution, leaving the distal end of the stem submerged 1.5 to 2.5 centimeters.	E9^(29)^, E10AD^(30)^, E11^(31)^, E18AD^(10)^, E21^(40)^
**10**	Fill the suction control chamber with water to a level of 20 centimeters of water (cmH2O).	E9^(29)^
**11**	Administer analgesia as prescribed by the doctor.	E2^(22)^, E7^(27)^
**12**	Assist in the procedure by opening the packages with aseptic technique, providing standardized antiseptic.	E17^(37)^, E21^(38)^
**13**	Record the volume of solution added to the collection bottle, as well as the date, time and name of the person responsible for the preparation.	E9^(29)^, E11^(31)^, E18AD^(10)^, E21^(40)^
**N°**	**Recommendations when inserting TD**	**Code of studies**
**14**	Non-drug pain control techniques should be considered.	E7^(27)^
**15**	Reassure the patient and monitor signs of discomfort, assessing the need to administer a new dose of prescribed analgesia and informing the medical team.	E4^(24)^, E7^(27)^, E21^(40)^
**16**	Support the patient during the procedure: advise them on the cold sensation of the germicidal solution and the sensation of pressure when infiltrating the local anesthetic.	E7^(27)^, E21^(40)^
**17**	Observe the patient carefully throughout the procedure, monitoring respiratory function, peripheral oxygen saturation and any hemodynamic changes.	E4^(24)^, E7^(27)^, E21^(40)^
**18**	Connect the drain to the previously filled collection bottle.	E7^(27)^, E21^(40)^
**19**	The connection must be fixed in such a way as to maintain visibility.	E11^(32)^, E16^(36)^
**20**	Connect the drain to suction when indicated and check that the system is working properly.	E7^(27)^
**21**	The patient’s skin around the insertion must be inspected and cleaned with aseptic technique.	E7^(27)^
**22**	Perform occlusive dressing according to the institution’s routine.	E7^(27)^, E21^(40)^
**N°**	**Recommendations after inserting TD**	**Code of studies**
**23**	Monitor vital signs every 2 hours, paying attention to breathing patterns, and assess and control pain using analgesics and non-drug techniques.	E4^(24)^, E7^(27)^, E9^(29)^, E17^(37)^, E18AD^(10)^, E21^(40)^
**24**	Keep the drainage bottle in an upright position below the level of the patient’s chest.	E1^(21)^, E3^(23)^, E4^(24)^, E7^(27)^, E9^(29)^, E10AD^(30)^, E14^(34)^, E17^(37)^, E18AD^(10)^, E19AD^(38)^, E21^(40)^
**25**	Assess and record the volume and appearance of the drains every 30 minutes to 1 hour for the first 3 to 4 hours after surgery.	E4^(24)^
**26**	Inform the medical team immediately if there is a sudden increase in blood drainage volume (drainage greater than 100ml/h), except in the first 3 hours after surgery.	E7^(27)^, E8^(28)^, E9^(29)^, E16^(36)^
**27**	Keep the drainage tube free of kinks, avoiding the formation of a dependent loop; if this is unavoidable, lift the tube every 15 minutes.	E1^(21)^, E3^(23)^, E4^(24)^, E7^(27)^, E8^(28)^, E9^(29)^, E10AD^(30)^, E16^(36)^, E18AD^(10)^
**28**	Observe for air infiltration around the tube insertion (subcutaneous emphysema).	E2^(22)^, E7^(27)^, E9^(29)^, E16^(36)^
**29**	Observe for fluid oscillation along the length of the drainage tube, indicating that the system is working properly.	E4^(24)^, E7^(27)^, E9^(29)^, E16^(36)^, E18AD^(10)^, E20AD^(39)^, E21^(40)^
**30**	Observe for bubbling, which should be slight, and notify the medical team if there is excessive or sudden bubbling.	E4^(24)^, E7^(27)^, E8^(28)^, E9^(29)^, E10AD^(30)^, E12AD^(32)^, E13^(33)^, E18AD^(10)^, E20AD^(39)^, E21^(40)^
**31**	Clamp the drains whenever the drainage bottle is positioned above the level of the patient’s chest, ensuring that the clamp remains in place for as short a time as possible.	E4^(24)^, E7^(27)^, E10AD^(30)^, E14^(34)^, E18AD^(10)^, E19AD^(38)^
**N°**	**Recommendations when inserting TD**	**Code of studies**
**32**	Do not clamp the drainage system even during patient transportation, just keep the system below the level of the patient’s chest.	E1^(21)^, E7^(27)^, E9^(29)^, E10AD^(30)^, E12AD^(32)^, E13^(33)^, E18AD^(10)^,
**33**	Clamp the system by hand for as short a time as possible (less than 1 minute), only to change the water seal, or clamp it with tweezers if the system is accidentally disconnected.	E1^(21)^, E4^(24)^, E5^(25)^, E8^(28)^, E10AD^(30)^, E13^(33)^,E14^(34)^, E18AD^(10)^, E19AD^(38)^, E20AD^(39)^
**34**	Discourage the use of clamps.	E5^(25)^, E9^(29)^, E10AD^(30)^, E13^(33)^, E18AD^(10)^, E19AD^(38)^
**35**	Change the water seal or drain bottle whenever there is a drain volume of more than 500 ml accumulated in the bottle.	E9^(29)^, E13^(33)^, E14^(34)^
**36**	Check the drainage rate (it should not exceed 200 mL/h in the first two to six hours).	E10AD^(30)^, E18AD^(10)^
**37**	Check drainage routinely (every 24 hours).	E9^(28)^, E10AD^(30)^, E13^(33)^, E14^(34)^, E20AD^(39)^
**38**	Make sure that the drain is connected to the correct inlet port on the collection bottle and, in the case of a suction drain, that the suction is turned on correctly.	E4^(24)^, E15^(36)^, E21^(40)^
**39**	Replenish the amount of fluid in the water seal bottle whenever necessary, ensuring that the stem is submerged between 1.5 and 3.0 centimeters or, in the suction bottle, 20 centimeters, as this volume will determine the pressure being applied to the suction.	E4^(24)^, E9^(29)^, E21^(40)^
**40**	Keep the vacuum pressure between 10 and 20 cmH2O.	E4^(24)^, E9^(29)^, E16^(36)^
**41**	Change the high-efficiency particulate air (HEPA) filter, indicated for patients in isolation due to the risk of aerosolization, such as SARS-CoV-2, per patient.	E16^(36)^
**42**	Educate the patient and family about what will be done, the care of the drainage system and the correct position in which the system should be held.	E7^(27)^, E9^(29)^, E14^(34)^, E17^(37)^, E18AD^(10)^, E21^(40)^
**43**	Encourage mobilization, coughing and deep breaths to facilitate drainage.	E4^(24)^, E7^(27)^, E9^(29)^, E18AD^(10)^, E20AD^(39)^, E21^(40)^
**44**	Change the dressing at the insertion site once a day, making sure it is clean, dry and free of odor and/or signs of infection.	E4^(24)^, E7^(27)^, E13^(33)^, E14^(34)^, E16^(36)^, E18AD^(10)^, E20AD^(39)^
**45**	Dress with aseptic technique, using gauze and 0.9% saline solution.	E4^(24)^, E9^(29)^, E14^(34)^, E18AD^(10)^
**46**	When available, use transparent film to apply the dressing, as this reduces the risk of infection and reduces the number of dressing changes, as the dressing can be kept on for three to seven days;	E17^(37)^, E21^(40)^
**47**	Apply a silicone-coated foam dressing with an adhesive border.	E15^(35)^
**48**	Inspect the drain insertion site daily for signs of infection and proper positioning.	E4^(24)^, E7^(27)^, E9^(29)^, E13^(33)^, E14^(34)^, E16^(36)^, E20AD^(39)^
**49**	Immediately cover the site with gauze and apply pressure to prevent negative inspiratory pressure from allowing air to enter the intrathoracic region if the chest tube is accidentally dislodged. Inform the doctor, organize material for reinserting the tube, reassure the patient and monitor them for signs of tension pneumothorax.	E12AD^(32)^
**50**	Massage the drain, if obstructed, with your hand to try to remove the clot or fibrin.	E3^(23)^, E4^(24)^, E9^(29)^, E10AD^(30)^
**51**	Discourage milking the drain.	E3^(23)^, E4^(24)^, E5^(25)^, E9^(29)^, E10AD^(30)^, E12AD^(32)^, E13^(33)^, E19AD^(38)^
**52**	Perform milking when requested by the doctor or to prevent obstruction.	E7^(27)^, E8^(28)^, E12AD^(32)^,E14^(34)^
**53**	Monitor for signs of resolution of pneumothorax, hemothorax, among others; by observing the volume and appearance of the drains.	E14^(34)^, E18AD^(10)^, E20AD^(39)^
**54**	Apply a cold compress 20 minutes before removing the chest tube to reduce the intensity of the pain associated with removing the tube.	E6^(26)^
**55**	Administer analgesia, as prescribed by the doctor, at least 30 minutes before removing the drain.	E1^(21)^, E4^(24)^, E6^(26)^
**56**	Instruct the patient on how to remove the chest tube and perform the Valsalva maneuver, as well as the procedure that will be carried out.	E2^(22)^, E9^(29)^
**57**	Prepare material for removing the drain and dressing.	E2^(22)^, E4^(24)^, E7^(27)^
**58**	Position the patient in a supine position with the arm raised above the head.	E2^(22)^
**59**	Apply an occlusive dressing after removing the drain.	E2^(22)^, E4^(24)^
**60**	Monitor the wound site for signs of infection and remove the stitches seven days after removing the drain.	E2^(22)^, E4^(24)^

## DISCUSSION

### Pre-Admission Recommendations

Double patient identification consists of implementing defenses and barriers, seeking to reduce the occurrence of incidents, ensuring that care is provided to the person for whom it is intended, protecting against risks and reducing the consequences of human failures or problems with equipment^(41)^. The use of resources that can identify the patient and adequately signal the allergic patient also seems to be in line with current patient safety and health risk management policies^(42)^.

Communication with the patient is an essential element that deserves attention from nurses, in order to ensure that the patient has no doubts. Implementing care strategies by providing educational material for patients can help reduce complications^(37)^.

In Brazil, the Informed Consent Form is usually applied by the doctor, as recommended by the Federal Council of Medicine^(43)^. However, it is up to the nurse to make sure that the patient has no doubts about what will be done and to make sure that the form has been duly signed^(37)^.

The preparation of appropriate material is fundamental to the organization of care^(37)^, so assembling the water seal system and, where appropriate, filling the suction bottle before inserting the drain, guarantees that the system will be ready for connection as soon as it is inserted. These recommendations consolidate the importance of nurses’ knowledge regarding the preparation of the chest drainage system. It should be emphasized that the level of suction applied to the suction drainage system is determined by the level of water in the bottles and not by the amount of vacuum applied. On the contrary, excessive bubbling, in addition to annoying the patient, can cause the fluid to evaporate more quickly and therefore reduce the level of suction. When connecting the drainage system to the suction system, slowly open the vacuum until a slight bubbling is observed in the suction bottle^(29)^.

Despite being a relatively simple procedure, TD can be painful. It is therefore up to the nursing team to administer appropriate analgesia, as prescribed by the doctor, in order to properly prepare the patient for the procedure^(27)^.

In addition, it is important to record the volume of solution added to the collection bottle, as well as the date, time and name of the person responsible for the preparation, in order to keep proper track of the volume drained and the period planned for changing the water seal^(29)^.

### Recommendations During Chest Tube Insertion

Current research shows that non-drug therapies, such as distraction techniques, music therapy, breathing techniques, acupuncture, among others, help to reduce pain and reduce the patient’s anxiety^(44,45)^. The importance of the nursing team’s role during the insertion of the chest tube should be emphasized. It is the nurse’s role to monitor signs of discomfort, hemodynamic aspects and the instructions that should be given during the procedure^(46,47)^.

Nurses should be responsible for handling the TD immediately after insertion, in accordance with Decree 94.406 of June 8, 1987, which states in Article 8: “nurses are responsible for: nursing care of greater technical complexity and requiring adequate scientific knowledge and the ability to make immediate decisions”^(47)^. In addition, Technical Chamber Opinion No. 22/2014/CTLN/COFEN, which addresses Good Practices in the care of chest drains, discusses that “nursing care with the chest drain includes various aspects relating to its insertion, handling, maintenance and removal”^(48)^. This care should therefore be carried out using the Systematization of Nursing Care (SNC), in order to reduce the risk of damage caused by negligence, malpractice or recklessness^(49)^.

### Recommendations After Chest Tube Insertion

There are a number of recommendations for care after insertion of the drain, ranging from monitoring the proper functioning of the system, maintaining permeability, proper positioning of the system, dressings and care during removal of the TD.

Careful and frequent monitoring helps in the early detection of complications^(46,47)^, considering that patients admitted to the Intensive Care Unit (ICU) are more likely to develop more aggressive complications related to the use of TD^(10,33)^.

Controlling the volume drained is essential for maintaining the patient’s hemodynamic stability, especially in the first three hours after surgery^(10)^. Studies^(33,50)^ suggest that drainage can be checked by marking the collection bottle, avoiding opening the system in order to reduce the risk of contamination. However, it is important to note that excessive accumulation of drainage (greater than 500 ml) in the collection bottle causes an increase in the system’s hydrostatic pressure, exceeding the transpulmonary gradient of expiration, making drainage difficult^(10,33)^ and potentially damaging the system’s permeability^(33)^.

Improper positioning of the system is one of the main ­non-standard practices in the care of TD systems^(19)^. Angles or folds in the system can, in addition to hindering drainage, facilitate the formation of clots and obstruction^(33)^.

Systematic assessment of fluid oscillation in the drainage tube, as well as the absence of air infiltration (subcutaneous emphysema) around the tube insertion, seem to be good indicators of system permeability^(51)^. These recommendations address precautions that should not only be used during the nurse’s routine assessment, but should also be well consolidated among the interdisciplinary team in order to detect possible complications as soon as possible^(51)^.

Some recommendations are controversial among the different studies, including those dealing with aspects related to clamping the system. It seems clear that clamping the system should be avoided as much as possible. However, if it is necessary, it should be done for as short a time as possible, using the hands and avoiding the use of clamps, so as not to run the risk of forgetting the occluded tube. In addition, it is not recommended to clamp the system for transportation, as clamping during pneumothorax treatment or in cases of air leakage can lead to increased intrapleural pressures, cardiac instability and the risk of tension pneumothorax in a short period of time^(10)^.

The creation of a checklist or tools that can support daily practice has had a positive impact on the qualification of care^(50)^. These tools can help nurses check items that may go unnoticed during the assessment, such as checking the fluid level in the collection bottles and/or the suction bottle, or the vacuum pressure being applied to the drainage system.

The use of a high-efficiency particulate air (HEPA) filter^(36)^ in the drainage system is specifically indicated for patients in isolation, owing to the risk of aerosolization, a strategy that was widely adopted during the SARS-CoV-2 pandemic. A small observational cohort study found that connecting two closed underwater drainage systems in series with an air filter connected to the second system was associated with a decrease in the spread of coronavirus particles^(52)^.

Another care course of action that showed a discrepancy between recommended practices is the most appropriate dressing, as well as the frequency with which it should be changed. It was observed that the most frequent recommendation is to use gauze and saline solution once a day or whenever necessary. At this point, the insertion site should be checked for signs of phlogiston and the device should be properly positioned^(38)^. However, it should be noted that dressing with transparent film has proven to be effective, as it reduces the risk of infection and the need for daily changes.

It is clear that mobilizing the patient facilitates drainage, highlighting the importance of interdisciplinary action between physiotherapy and the nursing team to encourage mobilization and proper positioning of the patient^(51)^. In addition, bed rest can increase the risk of deep vein thrombosis and embolism, and decrease intestinal peristalsis^(34)^.

If the chest tube is accidentally pulled, it is advisable to immediately cover the site with gauze and apply pressure to prevent negative inspiratory pressure from allowing air to enter the chest. In addition, the doctor should be informed immediately so that the tube can be reinserted^(53)^.

Nurses play a key role and have major responsibilities in all the stages involving the installation and maintenance of the TD. Therefore, monitoring the volume and characteristics of the fluid, controlling the level of suction, observing the permeability of the system and observing and controlling signs of infection have a direct impact on the recovery process^(37)^.

There seems to be a consensus among studies that patient and family education, with precise and easy guidance, reduces recovery time and increases positive outcomes. In addition, it is the nurse’s duty to educate the technical team in order to promote patient safety^(54)^.

There are also inconsistencies in the literature about milking, in which this practice is not widely recommended, especially as a method of preventing obstruction. In these cases maintenance of the drain in the correct position, avoiding the formation of angles that could cause fluid to accumulate in the system, seems to be sufficient^(33)^. This issue is also addressed in a contradictory way in Technical Chamber Opinion No. 22/2014/CTLN/COFEN, which states: “given the lack of scientific evidence to support the practice of milking the drainage system as a routine procedure to prevent the occurrence of obstruction, this practice should not be adopted. However, in the event of obstruction of the system, milking is necessary”^(48)^. One practice that is widely observed in daily practice is the use of pressure between the fingers of the hand on some segments of the drainage system, in an attempt to dislodge any clots or fibrin from the system, moving them to the collection bottle and enabling them to be removed, which has been shown to be a scientifically validated practice^(29,30)^.

One of the limitations of this study is the scarcity of studies addressing the care recommendations applicable to the drain removal process, despite the fact that they are frequently observed in daily practice. It is therefore suggested that more in-depth research be carried out into these practices, so that they can be widely implemented. In addition, the lack of consensus among the authors regarding some practices and the scarcity of studies with a high level of evidence that can support these practices are noteworthy.

## CONCLUSION

A total of 60 nursing care measures were mapped, 13 of which were applicable to the pre-insertion of the chest tube, nine during insertion and 38 after insertion. However, studies still address the lack of standardization of nursing actions in the care of patients with TD and emphasize the need for robust research that can support the implementation of EBP. This may be associated with the restriction on carrying out clinical trials in situations known to be harmful to patients.

The variables surrounding the problem encourage the importance of the link between academic institutions and hospitals for the purpose of qualified scientific production, in order to generate innovations and subsidies applicable to care. This review made it possible to trace the weakness of the evidence on the care provided to patients with TD, identifying gaps and topics of interest to be addressed in new studies. Among the gaps identified is the lack of recommendations for the angle of the head of the bed that should be maintained while the patient is using the TD. However, it is routinely observed that patients with respiratory dysfunctions are kept with the head elevated to 30° or 45°. In addition, there is a need for further studies to support the use of HEPA filters in patients in isolation due to the risk of aerosolization.
