# Supplementary material for: Nursing care for adult patients with chest drainage: a scoping review
Source: Rev Esc Enferm USP. 2024 Sep 30;58:e20240017. doi: 10.1590/1980-220X-REEUSP-2024-0017en (PMC11444167; doi:10.1590/1980-220X-REEUSP-2024-0017en)
Supplement: Translation

# Translation: Cuidados de enfermagem ao paciente adulto com drenagem torácica: uma revisão de escopo*

**DOI:** 10.1590/1980-220X-REEUSP-2024-0017pt

**Authors:** Elisiane Goveia da Silva, Bárbara Rodrigues Araujo, Raphaela de Matos Borges, Tainara Wink Vieira, Cristiane Cardoso de Paula, Rita Catalina Aquino Caregnato

**Affiliations:** 1 Universidade Federal de Ciências da Saúde de Porto Alegre, Departamento de Enfermagem, Porto Alegre, RS, Brasil.; 2 Hospital Nossa Senhora da Conceição, Porto Alegre, RS, Brasil.; 3 Universidade Federal de Santa Maria, Departamento de Enfermagem, Santa Maria, RS, Brasil.

**Keywords:** Cuidados de Enfermagem, Tubos Torácicos, Unidades de Terapia Intensiva

## Abstract

**Objetivo:**

Mapear os cuidados de enfermagem recomendados aos pacientes adultos submetidos à drenagem torácica.

**Método:**

Revisão de escopo. Incluíram-se estudos em português, inglês e espanhol, sem recorte temporal, que responderam à questão de pesquisa: quais são os cuidados de enfermagem indicados aos pacientes adultos com drenagem torácica internados em terapia intensiva? Seleção nas bases de dados *MEDLINE/PubMed*, *Embase/Elsevier*, *Web of Science/Clarivate*, *Scopus/Elsevier*, *CINAHL/Ebsco* e LILACS/BVS. Não foram incluídos dados de literatura cinzenta.

**Resultados:**

Dos 973 artigos identificados, selecionaram-se 21. Os cuidados mais citados incluíram: preencher frasco coletor com água destilada ou solução fisiológica deixando extremidade distal da haste submersa 1,5 a 2,5 centímetros; monitorização sinais vitais; manejo da dor; e posicionamento adequado do sistema. Observaram-se divergências na indicação de clampeamento do sistema e na ordenha do dreno/sistema de drenagem.

**Conclusão:**

Mapeados 60 cuidados de enfermagem, sendo 13 no momento pré-inserção do dreno, nove durante a inserção e 38 após a inserção do dreno de tórax.

## INTRODUÇÃO

A Prática Baseada em Evidências (PBE) visa melhorar a efetividade clínica e apoiar o profissional de saúde na tomada de decisão, adotando três elementos principais em sua abordagem: evidências científicas, experiência clínica e preferências do paciente^(1,2)^.

A atuação do enfermeiro na elaboração, aplicação e avaliação das tecnologias em saúde tem evoluído na produção de protocolos e materiais educacionais fundamentados na PBE. A Portaria n° 2.510/GM de 2005, define como tecnologias em saúde: medicamentos, materiais, equipamentos e procedimentos, sistemas organizacionais, educacionais, de informações e de suporte, programas e protocolos assistenciais, por meio dos quais a atenção e os cuidados com a saúde são prestados à população^(3)^.

As tecnologias utilizadas pela equipe de enfermagem para auxiliar as práticas diárias são classificadas em produtos e/ou processos. Definem-se produtos as informatizações, informações e artefatos; e os processos correspondem aos saberes estruturados, como as teorias e os instrumentos educacionais. Dessa forma, os protocolos assistenciais são considerados tecnologias em saúde, sendo indicados na organização dos processos de enfermagem e na prestação de cuidados adequados, de maneira segura e eficiente^(4)^.

Considerada uma tecnologia em saúde, a drenagem torácica (DT) consiste na instalação de um dreno tubular na cavidade pleural ligado a um sistema de selo d’água, com a finalidade de drenar o conteúdo anômalo do espaço pleural, visando a ­re-expansão pulmonar. Se necessário, um segundo frasco poderá ser acoplado ao sistema e conectado a uma rede de aspiração contínua, buscando aspirar de modo controlado e contínuo, ajudando a manter o equilíbrio da pressão negativa intratorácica^(5,6)^. A DT é considerada uma tecnologia segura e eficaz, muito utilizada em procedimentos eletivos e/ou de emergência no tratamento de complicações pulmonares, tais como: pneumotórax, hemotórax, derrame pleural complicado, empiema, quilotórax^(7)^ e no pós-operatório de cirurgias torácicas e mediastinais^(6)^.

Apesar de tratar-se de uma tecnologia comum no contexto hospitalar, a DT não está isenta de complicações. Um estudo descritivo, realizado no Brasil, com o objetivo de identificar os fatores preditores de complicações da DT em pacientes vítimas de trauma, apontou uma taxa de complicações de 26,3%^(8)^. Dentre as principais complicações associadas ao procedimento, pode-se citar: mau posicionamento do dreno, com necessidade de novo procedimento; hemotórax/pneumotórax residual; pneumonia e infecção; e permanência do orifício fora da cavidade torácica^(8)^.

A enfermagem atua em todas as etapas na assistência ao paciente, sendo primordial a implementação de cuidados baseados em evidências que promovam a segurança do paciente, com vistas à eficácia do tratamento e a prevenção de complicações^(9)^.

Pesquisadores do Hospital Universitário Semiurbano Nigeriano, que buscavam avaliar o nível de conhecimento sobre os cuidados com drenos torácicos entre enfermeiros, abordando desde aspectos anatômicos até os cuidados pós-procedimentos, evidenciaram que os profissionais de enfermagem acreditam na importância do treinamento sobre cuidados com dreno torácico, entretanto a maioria (66,7%) não recebeu capacitação para atualização sobre o assunto^(10)^. Além disso, cerca de 45% dos enfermeiros não sabiam ou não tinham certeza de que o nível de fluido flutuante na tubulação de drenagem era indicativo de bom funcionamento do dispositivo^(10)^. Sendo assim, torna-se imprescindível a produção de evidências científicas que possam subsidiar a construção de protocolos assistenciais de enfermagem ao paciente em uso da DT, promovendo a qualificação da assistência e tornando o cuidado mais seguro.

Frente a essa problemática, o estudo objetivou mapear os cuidados de enfermagem aplicados aos pacientes adultos submetidos à DT.

## MÉTODO

### Tipo do Estudo

Trata-se de uma revisão de escopo, seguindo as recomendações do Joanna Briggs Institute^(11,12)^, a qual é indicada para mapear conceitos e apresentar uma ampla visão das evidências pertencentes acerca de um determinado tópico, estratégia essa que demonstrou afinidade com o objetivo do estudo desenvolvido^(11,12)^. O protocolo desta revisão foi registrado na plataforma *Open Science Framework* (OSF) sob o DOI: 10.17605/osf.io/T8RW9 e previamente publicado^(13)^. Definiu-se a seguinte questão de revisão: “quais são os cuidados de enfermagem indicados aos pacientes adultos com DT internados em terapia intensiva?”.

Com o intuito de garantir a qualidade e transparência da redação, foi utilizado o guia para relatório de revisão: *Preferred Reporting Items for Systematic Reviews and Meta-Analyses Extension for Scoping Reviews (PRISMA-ScR) Checklist* ^(14,15)^.

### Critérios de Seleção

Foram incluídas publicações sem recorte temporal, estudos quantitativos, qualitativos, artigos de revisão, diretrizes clínicas e protocolos terapêuticos que atendem ao acrônimo PCC, sendo: P (população) os pacientes adultos com drenagem torácica; C (conceito) os cuidados de Enfermagem; e C (contexto) a terapia intensiva.

Foram excluídos editoriais, carta ao editor, artigos de opinião e revisões narrativas, pois apesar de possuírem um papel fundamental para a educação continuada, não possuem metodologia que permitam a reprodução dos dados e nem fornecem respostas para questões de pesquisa mais específicas^(16)^. Não foram avaliadas outras fontes de literatura cinzenta, pois ­considerou-se que a recorrência dos dados estaria indicando a saturação das informações^(17)^.

### Coleta de Dados

As estratégias de buscas foram construídas pelas pesquisadoras e revisadas por uma bibliotecária. Inicialmente, foi desenvolvida uma estratégia de busca para MEDLINE/PubMed e realizadas adaptações para as demais bases, a saber: *Embase*/*Elsevier*, *Web of Science*/*Clarivate*, *Scopus*/*Elsevier*, *Cumulative Index to Nursing and Allied Health Literature* (CINAHL)/*Ebsco* e Literatura Latino-Americana e do Caribe em Ciências da Saúde (LILACS)/Biblioteca Virtual em Saúde (BVS). Os termos de busca foram definidos a partir dos Descritores em Ciências da Saúde (DeCS)/*Medical Subject Headings* (MeSH) e descritores não controlados, combinados com uso de operadores booleanos *AND* e *OR* (Quadro 1).

**Quadro 1:** Termo de busca utilizado na base de dados MEDLINE/PubMed – Porto Alegre, RS, Brasil, 2023.

Base de dados	Estratégia
**MEDLINE/PubMed**	(Nursing Care[mh:noexp] OR Nursing[mh] OR Nursing, Team[mh] OR Nursing Staff, Hospital[mh] OR Nursing Service, Hospital[mh] OR Nursing Assessment[mh] OR Nursing Process[mh:noexp] OR Nurses[mh] OR Nursing[sh] OR Nursing*[tiab] OR Nurse*[tiab]) *AND* (Thoracostomy[mh] OR Thoracostom*[tiab] OR Thoracic drain*[tiab] OR Thorax drain*[tiab] OR Chest drain*[tiab] OR Pleural drain*[tiab] OR ((Chest Tubes[mh] OR Chest Tube*[tiab]) AND (Drainage[mh] OR Drain*[tiab] OR Suction[mh] OR Suction*[tiab])))
**Embase/Elsevier**	(‘nursing care’/exp OR ‘nursing’/exp OR ‘nursing staff’/exp OR ‘nurse’/exp OR Nursing*:ti,ab,kw OR Nurse*:ti,ab,kw) *AND* (‘thoracostomy’/exp OR ‘thorax drainage’/exp OR Thoracostom*:ti,ab,kw OR ‘Thoracic drain*’:ti,ab,kw OR ‘Thorax drain*’:ti,ab,kw OR ‘Chest drain*’:ti,ab,kw OR ‘Pleural drain*’:ti,ab,kw OR ((‘chest tube’/exp OR ‘Chest Tube*’:ti,ab,kw) AND (‘suction drain’/exp OR ‘drain’/de OR ‘suction’/de OR ‘suction drainage’/de OR Drain*:ti,ab,kw OR Suction*:ti,ab,kw))) *AND* *([embase]/lim NOT ([embase]/lim AND [medline]/lim))*
**Web of Science**	TS=(Nursing* OR Nurse*) *AND* TS=(Thoracostom* OR “Thoracic drain*” OR “Thorax drain*” OR “Chest drain*” OR “Pleural drain*”) OR (TS=”Chest Tube*” AND TS=(Drain* OR Suction*))
**Scopus**	TITLE-ABS-KEY(Nursing* OR Nurse*) *AND* (TITLE-ABS-KEY(Thoracostom* OR “Thoracic drain*” OR “Thorax drain*” OR “Chest drain*” OR “Pleural drain*”) OR (TITLE-ABS-KEY(“Chest Tube*”) AND TITLE-ABS-KEY(Drain* OR Suction*)))
**CINAHL**	(MH (“Nursing Care” OR Nursing OR “Nursing, Team” OR “Nursing Staff, Hospital” OR “Nursing Service, Hospital” OR “Nursing Assessment” OR “Nursing Process” OR “Nurses”) OR TI (Nursing* OR Nurse*) OR AB (Nursing* OR Nurse*) OR SU (Nursing* OR Nurse*)) *AND* (((MH Chest Tubes OR TI “Chest Tube*” OR AB “Chest Tube*” OR SU “Chest Tube*”) AND (MH (Drainage OR Suction) OR TI (Drain* OR Suction*) OR AB (Drain* OR Suction*) OR SU (Drain* OR Suction*))) OR MH thoracostomy OR TI (Thoracostom* OR “Thoracic drain*” OR “Thorax drain*” OR “Chest drain*” OR “Pleural drain*”) OR AB (Thoracostom* OR “Thoracic drain*” OR “Thorax drain*” OR “Chest drain*” OR “Pleural drain*”) OR SU (Thoracostom* OR “Thoracic drain*” OR “Thorax drain*” OR “Chest drain*” OR “Pleural drain*”)))
**BVS**	(mh:(“Nursing Care” OR Nursing OR “Nursing, Team” OR “Nursing Staff, Hospital” OR “Nursing Service, Hospital” OR “Nursing Assessment” OR “Nursing Process” OR N02.360.650*) OR ti:(Enfermage* OR Nursing* OR Enfermeria* OR Enfermera* OR Enfermero* OR Enfermeir*) OR ab:(Enfermage* OR Nursing* OR Enfermeria* OR Enfermera* OR Enfermero* OR Enfermeir*)) *AND* (mh:(“Chest Tubes” OR Thoracostomy) OR ti:(“Chest Tubes” OR “Chest Tube” OR “Tubos toracicos” OR “Tubo toracico” OR Thoracostom* OR Toracostom*) OR ab:(Thoracostom* OR Toracostom*) OR (tw:(Thorax* OR Thoracic* OR Torax OR Toracic* OR Pleura* OR Chest*) AND (tw:Drenage* OR Drenaje* OR Dreno* OR Drain* OR Suction* OR Succao OR Succoes OR Succion*)))

Os estudos foram importados no dia 29 de janeiro de 2023, em arquivos no formato para gerenciador de referências (RIS ou NBIB), para o *software Covidence* ^®^, ferramenta que auxilia na estratificação dos itens de acordo com cada etapa de seleção, atendendo às recomendações metodológicas deste estudo. A seleção dos estudos foi realizada por dois revisores independentes com experiência no tema, de forma cega. As divergências foram resolvidas em discussão entre os revisores, nos casos em que não foi estabelecido consenso um terceiro revisor foi consultado.

No primeiro momento houve a exclusão dos artigos identificados em duplicidade. Após, foram realizadas as leituras de títulos, resumos e artigos na íntegra, respectivamente. Além disso, em 19 de outubro de 2023, foram verificadas as listas de referências dos estudos selecionados, a fim de identificar publicações em potencial que não foram identificadas pela estratégia de busca inicial adotada e/ou publicações que possam ter ocorrido a *posteriori* da data inicial de importação dos estudos^(11)^.

### Análise e Tratamento dos Dados

Para extração dos dados, foi utilizado o *software Microsoft Excel^®^,* no qual foram caracterizados os estudos da amostra final, com as seguintes informações: título do estudo, ano de publicação, país de origem, objetivo, desenho e população. As recomendações foram extraídas em três subtemas temporais: pré, durante e após inserção do dreno de tórax. Os estudos foram identificados utilizando código sequencial E1 até E26 e, após a codificação sequencial, os adicionais com a sigla “AD”.

As revisões de escopo geralmente são conduzidas para fornecer uma visão geral das evidências existentes, independentemente da qualidade metodológica ou do risco de viés^(11)^. A análise dos dados extraídos foi de natureza descritiva. Os dados de caracterização dos estudos incluídos foram analisados com o uso de estatísticas descritivas. A seguir, estas recomendações foram descritas em sua especificidade^(18)^. Os resultados estão apresentados em quadros e resumo narrativo. A seção de discussão realiza a síntese das evidências encontradas durante a revisão, de forma a explorá-las e compará-las à literatura existente^(19)^.

### Aspectos Éticos

Foram respeitadas todas as exigências éticas e científicas fundamentais para a realização do estudo, baseadas nas orientações e disposições contidas na Lei n° 12.853, de 14 de agosto de 2013, que dispõe sobre a gestão coletiva de direitos autorais e dá outras providências, assegurando a autoria dos estudos utilizados, bem como a autenticidade de ideias, conceitos e definições dos autores, com o intuito de preservar os direitos autorais^(20)^.

## RESULTADOS

O processo de seleção iniciou com 973 estudos mapeados nas bases de dados, desses foram selecionados 21 estudos para extração das recomendações. Dentre os estudos incluídos, nos que constam estudos em português, inglês e espanhol, cinco foram adicionados pela busca manual, através da avaliação das listas de referências, por responderem à questão de revisão (Figura 1). Destaca-se que dentre os estudos excluídos, houve um total de 110 estudos que não foram encontrados os textos completos para avaliação, mesmo após tentativa de contato com os autores.

**Figura 1:** Fluxograma da seleção dos estudos – Porto Alegre, RS, Brasil, 2023^(15)^.

Dentre os estudos selecionados, oito são revisões de literatura, oito estudos transversais observacionais, duas revisões sistemáticas, dois estudos randomizados e um estudo de validação de conteúdo. Quanto à origem, sete foram produzidos na América do Sul, três nos Estados Unidos, três na Europa, três no Oriente Médio, dois no Egito, dois na Ásia e um na Austrália (Quadro 2).

**Quadro 2:** Caracterização dos estudos selecionados – Porto Alegre, RS, Brasil, 2023.

Código	Título do Estudo	Ano	País de Ori-gem	Objetivo do Estudo	Desenho	População
E1	Nursing management of chest drains: a systematic review^(21)^	2001	Austrá-lia	Apresentar a melhor evidência de pesquisa disponível sobre o manejo de enfermagem de drenos torácicos.	Revisão sistemática	Oito ensaios controlados randomizados.
E2	Retirada de dreno torácico em pós-operatório de cirurgia cardíaca^(22)^	2005	Brasil	Elaborar um roteiro para retirada de dreno torácico por enfermeiros em unidade coronariana e cirurgia cardíaca.	Revisão bibliográfica	Não consta número total de estudos incluídos.
E3	To strip or not to strip? Physiological effects of chest tube manipulation^(23)^	2007	Estados Unidos	Resumir as evidências científicas em relação a ordenha dos drenos torácicos.	Revisão de literature	Cinco estudos de pesquisa originais, uma revisão sistemática, dois estudos descritivos exploratórios.
E4	Nursing management of patients with a chest drain^(24)^	2008	Reino Unido	Examinar o papel da enfermagem no gerenciamento da drenagem torácica, desde a inserção até a remoção, e incluir aspectos do controle da dor características de um dreno torácico funcional.	Revisão de literature	Entre 2000-2007, 15 artigos; porém, os autores aumentaram o período para 1995-2007 e não informaram total de artigos.
E5	A influência do uso do “clamp” ou braçadeira no acúmulo de coágulos em drenos pleurais tubulares^(25)^	2008	Brasil	Avaliar a influência do uso da braçadeira sobre o acúmulo de coágulos dentro dos drenos pleurais.	Estudo de campo prospectivo	53 pacientes (53 drenos pleurais).
E6	The Effect of Cold Application in Combination with Standard Analgesic Administration on Pain and Anxiety during Chest Tube Removal: A Single-Blinded, Randomized, Double-Controlled Study^(26)^	2010	Turquia	Investigar o efeito da aplicação de frio na dor e ansiedade durante a remoção do dreno torácico no paciente submetido a cirurgia cardíaca.	Estudo randomizado simples-cego	90 pacientes internados na UTI cirúrgica cardiovascular e torácica.
E7	Assistência de enfermagem na drenagem torácica: revisão de literatura^(27)^	2011	Brasil	Realizar revisão de literatura sobre a assistência de enfermagem na drenagem torácica.	Revisão de literature	Não consta número total de estudos incluídos.
E8	Analytical Study on Practices Related to Care of Water Sealed Chest Drainage System^(28)^	2012	Índia	Analisar as práticas prevalentes no cuidado do sistema de drenagem torácica selado com água de acordo com as opiniões dos especialistas e contra as evidências disponíveis.	Estudo observacional	14 especialistas.
E9	Chest tube care in critically ill patient: A comprehensive review^(29)^	2015	Egito	Conhecer riscos associados à inserção do dreno torácico. Descrever forma de preparo de unidade de drenagem torácica, realizar avaliações contínuas do paciente, documentar adequadamente e solucionar possíveis problemas relacionados ao uso de dreno torácico.	Revisão de literature	Não consta número total de estudos incluídos.
E10AD	Manipulação de drenos mediastinais e pleurais: existe evidência científica?^(30)^	2015	Brasil	Verificar a existência de evidência científica na manipulação de drenos pleurais e mediastinais na cirurgia cardíaca.	Revisão sistemática	Seis artigos, sendo uma revisão, um ensaio clínico controlado, um estudo experimental controlado e dois estudos descritivos.
E11	Institutional protocol to standardize the chest drainage system management, from surgery to nursing care, at a regional hospital in northern Paraná^(31)^	2016	Brasil	Avaliar o manejo dos sistemas fechados de drenagem torácica por meio da análise de pacientes adultos do Hospital Universitário Regional de Maringá, Paraná, padronizar o protocolo de atendimento em drenagem torácica e minimizar complicações.	Estudo observacional prospectivo	Observadas 100 drenagens torácicas, de 83 pacientes.
E12AD	Nurses’ knowledge and Practice regard Care of Patient with Chest Drains in Sudan Heart Center, Khartoum, Sudan^(32)^	2016	Sudão	Avaliar o conhecimento e a prática do enfermeiro em relação ao paciente conectado à drenagem torácica.	Estudo quantitativo descritivo	50 enfermeiros.
E13	Variation in nurse self-reported practice of managing chest tubes: A cross-sectional study^(33)^	2018	China	Revelar a prática autorrelatada de gerenciamento de drenos torácicos por enfermeiras e definir os tomadores de decisão para essas práticas.	Estudo transversal	296 enfermeiros.
E14	Intervenção de enfermagem: cuidados com dreno torácico em adultos no pós-operatório^(34)^	2018	Brasil	Validar as atividades de enfermagem para intervenção estabelecida pela *Nursing Interventions Classification* “cuidados com dreno torácico”.	Estudo de validação de conteúdo	30 enfermeiras especialistas.
E15	Comparative Evaluation of Chest Tube Insertion Site Dressings: A Randomized Controlled Trial^(35)^	2019	Estados Unidos	Comparar vários tipos de curativos e procedimentos após a colocação de drenos torácicos e mediastinais.	Estudo prospectivo randomizado	127 pacientes, de três Unidades de Terapia Intensiva, e 236 drenos de tórax. Amostra três grupos, cada um com uma cobertura e frequência de troca diferentes.
E16	Evidence-based management of patients with chest tube drainage system to reduce complications in cardiothoracic vascular surgery wards^(36)^	2021	Estados Unidos	Implementar as melhores práticas para fornecer cuidados seguros e eficazes a pacientes com sistema de drenagem torácica em enfermarias cardiotorácicas.	Revisão de literature	Não consta número de estudos incluídos.
E17	Interdisciplinary teamwork for chest tube insertion and management: an integrative review^(37)^	2021	França	Identificar os artigos relacionados ao manejo interdisciplinar da toracotomia.	Revisão integrative	19 artigos.
E18AD	Boas práticas de enfermagem na utilização de dreno de tórax: revisão integrativa^(10)^	2021	Brasil	Identificar na literatura científica as melhores práticas de enfermagem relacionadas à utilização de dreno torácico em Unidade de Terapia Intensiva adulta.	Revisão integrative	Cinco artigos, sendo um quanti-qualitati-vo e quatro quantitativos.
E19AD	Chest drains: prevalence of insertion and ICU nurses’ knowledge of care^(38)^	2021	Jordâ-nia	Descrever a taxa de prevalência de inserção de dreno torácico nas UTIs jordanianas e avaliar o nível de conhecimento dos enfermeiros jordanianos sobre os cuidados com o dreno torácico.	Estudo descritivo não experimental com pesquisa transversal	1973 arquivos analisados e questionário com 225 enfermeiros.
E20AD	Nurses’ Knowledge Levels About the Care of the Patients with Chest Tube^(39)^	2021	Turquia	Determinar o nível de conhecimento dos enfermeiros sobre os cuidados aos pacientes com dreno torácico.	Estudo descritivo	152 enfermeiros.
E21	Development and validation of the first performance assessment scale for interdisciplinary chest tube insertion: a prospective multicenter study^(40)^	2022	Alema-nha	Validar uma escala interdisciplinar de avaliação de desempenho para inserção de dreno torácico desenvolvida a partir da análise da literatura.	Estudo multicêntrico prospectivo	40 vídeos de sessões de simulação realizadas por 80 participantes.

Foram extraídas um total de 60 recomendações, sendo divididas em 13 pré, nove durante e 38 após inserção da DT. A totalidade das recomendações foram organizadas conforme os respectivos estudos, fonte da informação e o número total de estudos que contemplam cada item (Quadro 3).

**Quadro 3:** Recomendações extraídas da amostra final – Porto Alegre, RS, Brasil, 2023.

N°	Recomendações pré-inserção do DT	Código dos estudos
**1**	Verificar dupla identificação do paciente.	E21^(40)^
**2**	Informar o paciente sobre o procedimento e como ele poderá ajudar.	E7^(27)^, E17^(37)^, E21^(40)^
**3**	Orientar a família sobre o cuidado adequado.	E14^(34)^
**4**	Aplicar Termo de Consentimento Informado.	E7^(27)^, E17^(37)^, E21^(40)^
**5**	Verificar alergias.	E7^(27)^, E21^(40)^
**6**	Preparar os materiais adequados para inserção do DT: solução antisséptica (sugere-se o uso de solução de clorexidine alcoólica pelo maior custo-benefício), avental, máscara e luvas estéreis, campos estéreis, bandeja para tubo torácico esterilizada, anestésico local, agulhas, seringas, bisturi com lâmina, pinças curvas kelly, dreno torácico, sistema de drenagem, lixo perfurocortante, material para o curativo.	E17^(37)^, E21^(40)^
**7**	Higienização simples das mãos com antisséptico.	E14^(34)^, E21^(40)^, E18AD^(10)^
**8**	Posicionar o paciente no leito confortavelmente: com o braço do lado onde o dreno será inserido, colocado atrás da cabeça de modo a expor a axila. O cirurgião poderá solicitar um travesseiro ou cobertor dobrado sob as omoplatas do paciente para elevar o tórax e facilitar o acesso.	E4^(24)^, E7^(27)^, E9^(29)^, E21^(40)^
**9**	Preencher o frasco coletor com água destilada ou solução fisiológica deixando a extremidade distal da haste submersa de 1,5 a 2,5 centímetros.	E9^(29)^, E10AD^(30)^, E11^(31)^, E18AD^(10)^, E21^(40)^
**10**	Preencher a câmara de controle de sucção com água até o nível de 20 centímetros de água (cmH2O).	E9^(29)^
**11**	Administrar analgesia conforme prescrição médica.	E2^(22)^, E7^(27)^
**12**	Auxiliar no procedimento abrindo as embalagens com técnica asséptica, fornecendo antisséptico padronizado.	E17^(37)^, E21^(38)^
**13**	Registrar volume de solução adicionado no frasco coletor, bem como, data, hora e nome do responsável pelo preparo.	E9^(29)^, E11^(31)^, E18AD^(10)^, E21^(40)^
**N°**	**Recomendações durante inserção do DT**	**Código dos estudos**
**14**	Técnicas de controle da dor não medicamentosas devem ser consideradas.	E7^(27)^
**15**	Tranquilizar o paciente e monitorar sinais de desconforto, avaliando a necessidade de administrar nova dose de analgesia prescrita e informando a equipe médica.	E4^(24)^, E7^(27)^, E21^(40)^
**16**	Apoiar o paciente durante o procedimento: orientá-lo quanto a sensação de frio da solução germicida e para a sensação de pressão quando infiltração do anestésico local.	E7^(27)^, E21^(40)^
**17**	Observar atentamente o paciente durante todo o procedimento, monitorando a função respiratória, saturação periférica de oxigênio e quaisquer alterações hemodinâmicas.	E4^(24)^, E7^(27)^, E21^(40)^
**18**	Conectar o dreno ao frasco coletor previamente preenchido.	E7^(27)^, E21^(40)^
**19**	A conexão deve ser fixada de forma a manter a visibilidade.	E11^(32)^,E16^(36)^
**20**	Ligar o dreno em aspiração quando indicado e verificar o funcionamento adequado do sistema.	E7^(27)^
**21**	A pele do paciente ao redor da inserção deve ser inspecionada e limpa com técnica asséptica.	E7^(27)^
**22**	Realizar curativo oclusivo conforme rotina da instituição.	E7^(27)^, E21^(40)^
**N°**	**Recomendações após inserção do DT**	**Código dos estudos**
**23**	Monitorar os sinais vitais a cada 2 horas, com atenção ao padrão respiratório, e avaliar e controlar a dor com o uso de analgésicos e aplicação de técnicas não medicamentosas.	E4^(24)^, E7^(27)^, E9^(29)^, E17^(37)^, E18AD^(10)^, E21^(40)^
**24**	Manter o frasco de drenagem em posição vertical abaixo do nível do tórax do paciente.	E1^(21)^, E3^(23)^, E4^(24)^, E7^(27)^, E9^(29)^, E10AD^(30)^, E14^(34)^, E17^(37)^, E18AD^(10)^, E19AD^(38)^, E21^(40)^
**25**	Avaliar e registrar o volume e aspecto das drenagens a cada 30 minutos a 1 hora nas primeiras 3 a 4 horas após a cirurgia.	E4^(24)^
**26**	Informar a equipe médica imediatamente se houver aumento repentino do volume de drenagem de sangue (drenagem maior que 100ml/h), exceto nas 3 primeiras horas após a cirurgia.	E7^(27)^, E8^(28)^, E9^(29)^, E16^(36)^
**27**	Manter a tubulação de drenagem livre de dobras, evitando formação de loop dependente, caso seja inevitável, levantar o tubo a cada 15 minutos.	E1^(21)^, E3^(23)^, E4^(24)^, E7^(27)^, E8^(28)^, E9^(29)^, E10AD^(30)^, E16^(36)^, E18AD^(10)^
**28**	Observar infiltração de ar ao redor da inserção do tubo (enfisema subcutâneo).	E2^(22)^, E7^(27)^, E9^(29)^, E16^(36)^
**29**	Observar se há oscilação do fluído na extensão do tubo de drenagem, indicando o bom funcionamento do sistema.	E4^(24)^, E7^(27)^, E9^(29)^, E16^(36)^, E18AD^(10)^, E20AD^(39)^, E21^(40)^
**30**	Observar borbulhamento que deve ser leve e comunicar à equipe médica se houver borbulhamento excessivo ou repentino.	E4^(24)^,E7^(27)^, E8^(28)^, E9^(29)^, E10AD^(30)^, E12AD^(32)^, E13^(33)^, E18AD^(10)^, E20AD^(39)^, E21^(40)^
**N°**	**Recomendações após inserção do DT**	**Código dos estudos**
**31**	Clampear os drenos sempre que o frasco de drenagem estiver posicionado acima do nível do tórax do paciente, assegurando que o clampe fique no local pelo menor tempo possível.	E4^(24)^, E7^(27)^, E10AD^(30)^, E14^(34)^, E18AD^(10)^, E19AD^(38)^
**32**	Não clampear o sistema de drenagem mesmo durante o transporte do paciente, apenas deve-se manter o sistema abaixo do nível do tórax do paciente.	E1^(21)^, E7^(27)^, E9^(29)^, E10AD^(30)^, E12AD^(32)^, E13^(33)^, E18AD^(10)^,
**33**	Clampear o sistema com a mão, pelo menor tempo possível (menos de 1 minuto), apenas para troca do selo d’água, ou clampeado com pinças se houver desconexão acidental do sistema.	E1^(21)^, E4^(24)^, E5^(25)^, E8^(28)^, E10AD^(30)^, E13^(33)^,E14^(34)^, E18AD^(10)^, E19AD^(38)^, E20AD^(39)^
**34**	Desencorajar o uso de *clamp* ou braçadeira.	E5^(25)^, E9^(29)^, E10AD^(30)^, E13^(33)^, E18AD^(10)^, E19AD^(38)^
**35**	Trocar o selo d’água ou o frasco de drenagem sempre que houver volume de drenagem maior que 500 ml acumulada no frasco.	E9^(29)^, E13^(33)^, E14^(34)^
**36**	Verificar a medida de drenagem (esta não deve exceder 200 mL/h nas primeiras duas a seis horas).	E10AD^(30)^, E18AD^(10)^
**37**	Verificar a drenagem rotineiramente (a cada 24 horas).	E9^(28)^, E10AD^(30)^, E13^(33)^, E14^(34)^, E20AD^(39)^
**38**	Certificar de que o dreno esteja conectado na porta correta de entrada do frasco coletor, bem como, em caso de dreno em aspiração, se a aspiração está ligada corretamente.	E4^(24)^, E15^(36)^, E21^(40)^
**39**	Providenciar reposição, sempre que preciso, da quantidade de fluídos no frasco de selo d’água, garantido que a haste fique submersa entre 1,5 e 3,0 centímetros ou, no frasco de aspiração, 20 centímetros; pois este volume que determinará a pressão que está sendo aplicada de aspiração.	E4^(24)^, E9^(29)^, E21^(40)^
**40**	Manter a pressão de vácuo entre 10 e 20 cmH2O.	E4^(24)^, E9^(29)^, E16^(36)^
**41**	Realizar troca de filtro de ar particulado de alta eficiência (HEPA), indicada nos pacientes em isolamento por risco de aerossolização, como SARS-CoV-2, por paciente.	E16^(36)^
**42**	Orientar o paciente e a família sobre o que será feito, os cuidados com o sistema de drenagem e a posição correta em que o sistema deve ser mantido.	E7^(27)^,E9^(29)^, E14^(34)^, E17^(37)^, E18AD^(10)^, E21^(40)^
**43**	Incentivar mobilização, tosse, e inspirações profundas para facilitar a drenagem.	E4^(24)^,E7^(27)^, E9^(29)^, E18AD^(10)^, E20AD^(39)^, E21^(40)^
**44**	Realizar troca de curativo no local de inserção uma vez ao dia, observando se o local está limpo, seco e sem odor e/ou sinais de infecção.	E4^(24)^, E7^(27)^, E13^(33)^, E14^(34)^, E16^(36)^, E18AD^(10)^, E20AD^(39)^
**45**	Realizar o curativo com técnica asséptica, podendo ser feito com gaze e solução fisiológica 0,9%.	E4^(24)^, E9^(29)^, E14^(34)^, E18AD^(10)^
**46**	Usar, quando disponível, película transparente para realização do curativo, pois diminui o risco de infecções e diminui as trocas pois o curativo pode ser mantido de três a sete dias;	E17^(37)^, E21^(40)^
**47**	Realizar a aplicação de curativo de espuma revestido com silicone com borda adesiva.	E15^(35)^
**48**	Inspecionar o local de inserção do dreno diariamente, observando a presença de sinais de infecção e o posicionamento adequado.	E4^(24)^, E7^(27)^, E9^(29)^, E13^(33)^, E14^(34)^, E16^(36)^, E20AD^(39)^
**49**	Cobrir imediatamente o local com gaze e aplicar pressão para evitar que a pressão inspiratória negativa permita a entrada de ar na região intratorácica, caso o dreno torácico seja desalojado acidentalmente. Informar o médico, organizar material para reinserção do tubo, tranquilizar o paciente e monitorá-lo quanto a sinais de pneumotórax hipertensivo.	E12AD^(32)^
**50**	Massagear o dreno, caso esteja obstruído, com a mão para tentar remover o coágulo ou fibrina.	E3^(23)^, E4^(24)^, E9^(29)^, E10AD^(30)^
**51**	Desencorajar a ordenha do dreno.	E3^(23)^, E4^(24)^, E5^(25)^, E9^(29)^, E10AD^(30)^, E12AD^(32)^, E13^(33)^, E19AD^(38)^
**52**	Realizar ordenha quando solicitado pelo médico ou para prevenir obstrução.	E7^(27)^, E8^(28)^, E12AD^(32)^,E14^(34)^
**53**	Monitorar quanto aos sinais de resolução do pneumotórax, hemotórax, entre outros; através da observação do volume e aspecto das drenagens.	E14^(34)^, E18AD^(10)^, E20AD^(39)^
**54**	Aplicar compressa fria 20 minutos antes da retirada do dreno torácico para redução da intensidade da dor associada a retirada do tubo.	E6^(26)^
**55**	Administrar analgesia, conforme prescrição médica, no mínimo 30 minutos antes da retirada do dreno.	E1^(21)^, E4^(24)^, E6^(26)^
**56**	Instruir o paciente para retirada do dreno torácico, a realizar a manobra de Valsalva, bem como sobre o procedimento que será realizado.	E2^(22)^, E9^(29)^
**57**	Preparar material para retirada do dreno e curativo.	E2^(22)^, E4^(24)^, E7^(27)^
**58**	Posicionar o paciente em posição dorsal com o braço elevado sobre a cabeça.	E2^(22)^
**59**	Realizar curativo oclusivo após a retirada do dreno.	E2^(22)^, E4^(24)^
**60**	Monitorar local da ferida quanto aos sinais de infecção e retirar os pontos sete dias após a retirada do dreno.	E2^(22)^, E4^(24)^

## DISCUSSÃO

### Recomendações Pré-Inserção

A dupla identificação do paciente consiste na implementação de defesas e barreiras, buscando reduzir a ocorrência de incidentes, assegurando que o cuidado será prestado à pessoa a qual se destina, relaciona proteção contra riscos e a redução das consequências provenientes de falhas humanas ou advindas de problemas com equipamentos^(41)^. A utilização de recursos que possam identificar o paciente e sinalizar de forma adequada o paciente alérgico, parece também estar em consonância com as políticas atuais de segurança do paciente e gestão de riscos em saúde^(42)^.

A comunicação com o paciente é um elemento essencial que merece atenção dos enfermeiros, visando garantir que o paciente não tenha dúvidas. A implementação de estratégias de cuidado, por meio do fornecimento de material educativo para os pacientes, pode contribuir para a redução das complicações^(37)^.

No Brasil, usualmente, o Termo de Consentimento Informado é aplicado pelo médico, conforme recomendação do Conselho Federal de Medicina^(43)^. Porém, cabe ao enfermeiro certificar-se de que o paciente não tenha dúvidas sobre o que será realizado e certificar-se de que o termo foi devidamente assinado^(37)^.

O preparo de material adequado é fundamental para organização do cuidado^(37)^, portanto, a montagem do sistema de selo d’água e, quando for o caso, o preenchimento do frasco de sucção antecipadamente à inserção do dreno, garante que o sistema esteja pronto para conexão assim que esse for inserido. Essas recomendações consolidam a importância do conhecimento do enfermeiro quanto ao preparo do sistema de drenagem torácica. Enfatiza-se que o nível de sucção, aplicado no sistema de drenagem em aspiração, é determinado pelo nível de água nos frascos e não pela quantidade de vácuo aplicada. Pelo contrário, o borbulhar excessivo, além de incomodar o paciente, pode causar a evaporação mais rápida do fluido e, portanto, diminuir o nível de sucção. Ao conectar o sistema de drenagem no sistema de aspiração, deve-se abrir lentamente o vácuo até se observar um leve borbulhar no frasco de sucção^(29)^.

Apesar de ser um procedimento relativamente simples, a DT pode ser dolorosa. Dessa forma, cabe à equipe de enfermagem administrar a analgesia adequada, conforme prescrição médica, visando preparar corretamente o paciente para o procedimento^(27)^.

Ademais, o registro do volume de solução adicionado no frasco coletor, bem como data, hora e nome do responsável pelo preparo é importante para obter um controle adequado do volume drenado e do período previsto para troca do selo d’água^(29)^.

### Recomendações Durante a Inserção do Dreno de Tórax

Pesquisas atuais abordam que terapias não medicamentosas, como técnicas de distração, musicoterapia, técnicas de respiração, acupuntura, entre outras, ajudam a reduzir a dor e diminuem a ansiedade do paciente^(44,45)^. Ressalta-se a importância da atuação da equipe de enfermagem durante a inserção do dreno de tórax. É papel do enfermeiro a monitorização de sinais de desconforto, dos aspectos hemodinâmicos e as orientações que devem ser realizadas durante o procedimento^(46,47)^.

Os cuidados com a manipulação da DT imediatamente após a inserção deverão ser realizados por enfermeiros, conforme o decreto n° 94.406 de 8 de junho de 1987, que aborda no Art. 8°: “ao enfermeiro incumbe: os cuidados de enfermagem de maior complexidade técnica e que exijam conhecimentos científicos adequados e capacidade de tomar decisões imediatas”^(47)^. Além disso, o Parecer de Câmara Técnica n° 22/2014/CTLN/COFEN, que aborda as Boas Práticas no cuidado com drenos de tórax, estabelece que “os cuidados de enfermagem com o dreno de tórax compreendem diversos aspectos relativos à sua inserção, manipulação, manutenção e retirada”^(48)^. Esses cuidados devem, portanto, ser empregados mediante a Sistematização da Assistência de Enfermagem (SAE), buscando reduzir riscos de danos causados por negligência, imperícia ou imprudência^(49)^.

### Recomendações Após a Inserção do Dreno de Tórax

Diversos são os cuidados elencados para após inserção do dreno, os quais envolvem desde a monitorização do funcionamento adequado do sistema, manutenção da permeabilidade, posicionamento adequado do sistema, até os curativos e cuidados durante a remoção da DT.

A monitorização criteriosa e frequente auxilia na detecção precoce de complicações^(46,47)^, considerando que os pacientes internados em Unidade de Terapia Intensiva (UTI) apresentam maior propensão a desenvolverem complicações mais agressivas relacionadas ao uso da DT^(10,33)^.

O controle de volume drenado é fundamental para manutenção da estabilidade hemodinâmica do paciente, principalmente nas três primeiras horas após a cirurgia^(10)^. Estudos^(33,50)^ orientam que a drenagem pode ser verificada através da marcação no frasco coletor, evitando abrir o sistema, a fim de reduzir o risco de contaminações. Contudo, é importante destacar que o acúmulo excessivo de drenagem (maior que 500 ml) no frasco coletor provoca aumento da pressão hidrostática do sistema, superando o gradiente transpulmonar da expiração, dificultando a drenagem^(10,33)^ e podendo prejudicar a permeabilidade do sistema^(33)^.

O posicionamento inadequado do sistema está entre as principais práticas não padronizadas nos cuidados com sistemas de DT^(19)^. As angulações ou dobras no sistema podem, além de dificultar a drenagem, facilitar a formação de coágulos e obstrução^(33)^.

A avaliação sistemática da oscilação do fluído no tubo de drenagem, assim como, da ausência de infiltração de ar (enfisema subcutâneo) ao redor da inserção do tubo, parecem ser bons indicadores de permeabilidade do sistema^(51)^. Essas recomendações abordam cuidados que devem ser empregados não apenas durante a avaliação rotineira do enfermeiro, mas que devem estar bem consolidadas entre a equipe interdisciplinar com o intuito da detecção breve de possíveis complicações^(51)^.

Algumas recomendações mostram-se controversas entre os diferentes estudos, dentre essas, as que tratam dos aspectos relacionados ao clampeamento do sistema. Parece estar claro que o clampeamento do sistema deve ser evitado ao máximo. Porém, se for necessário realizá-lo, deve-se executar pelo menor tempo possível, utilizando as mãos e evitando o uso de *clamps* ou braçadeiras, para não incorrer no risco de esquecer o tubo ocluído. Além disso, não é recomendado o clampeamento do sistema para a realização de transporte, visto que o clampeamento em tratamento de pneumotórax ou nos casos com escape de ar, pode gerar, em curto período de tempo, o aumento das pressões intrapleurais, instabilidade cardíaca e risco para pneumotórax hipertensivo^(10)^.

A criação de um *checklist* ou instrumentos que possam apoiar a prática diária tem demonstrado impacto positivo na qualificação do cuidado^(50)^. Essas ferramentas podem auxiliar o enfermeiro na verificação de itens que possam passar despercebido durante a avaliação, como por exemplo, a verificação do nível de fluido nos frascos coletor e/ou no frasco de aspiração ou, ainda, a pressão de vácuo que está sendo aplicada ao sistema de drenagem.

A utilização de filtro de ar particulado de alta eficiência (HEPA)^(36)^ no sistema de drenagem está indicada especificamente para pacientes em isolamento por risco de aerossolização, estratégia bastante adotada durante a pandemia da SARS-CoV-2. Um pequeno estudo observacional de coorte descobriu que a ligação de dois sistemas de drenagem subaquática fechados, em série com um filtro de ar conectado ao segundo sistema, estava associada a uma diminuição na disseminação de partículas do coronavírus^(52)^.

Outro cuidado que demonstrou discrepância entre as práticas recomendadas, trata do curativo mais adequado, bem como, a frequência em que esse deverá ser trocado. Observou-se que a recomendação mais frequente consiste na utilização de gaze e solução fisiológica, uma vez ao dia ou sempre que necessário. Nesse momento deve-se observar o local de inserção quanto a presença de sinais flogísticos, bem como, do posicionamento adequado do dispositivo^(38)^. Contudo, ressalta-se que o curativo com aplicação de película transparente, demonstrou ser eficaz, pois reduz o risco de infecções e a necessidade de troca diária.

Evidencia-se que mobilizar o paciente facilita a drenagem, ressaltando a importância da atuação interdisciplinar entre a fisioterapia e a equipe de enfermagem para o incentivo à mobilização e posicionamento adequado do paciente^(51)^. Além disso, a restrição no leito pode promover aumento do risco de trombose venosa profunda e embolia, e diminuição do peristaltismo intestinal^(34)^.

Caso o dreno torácico seja tracionado acidentalmente, recomenda-se cobrir imediatamente o local com gaze e aplicar pressão para evitar que a pressão inspiratória negativa permita a entrada de ar para o tórax. Além disso, o médico deverá ser imediatamente informado para possível reinserção do tubo^(53)^.

O enfermeiro desempenha papel fundamental e com grandes responsabilidades em todas as etapas que envolvem a instalação e manutenção da DT. Sendo assim, a monitorização do volume e características do fluido, o controle do nível de sucção, a observação da permeabilidade do sistema e a observação e controle de sinais de infecção impactam diretamente no processo de recuperação^(37)^.

Parece existir um consenso entre os estudos de que a educação do paciente e da família, com orientações precisas e fáceis, reduz o tempo de recuperação e aumenta os resultados positivos. Além disso, caracteriza-se dever do enfermeiro promover educação da equipe técnica, buscando promoção da segurança do paciente^(54)^.

Observa-se ainda, inconsistências na literatura acerca da realização da ordenha, na qual essa prática não é amplamente recomendada, principalmente como método para prevenção de obstrução. Nesses casos, a manutenção do dreno em posição correta, evitando formação de angulações que possam gerar acúmulo de líquido no sistema, parece ser suficiente^(33)^. Esse tema também é abordado de forma contraditória no Parecer de Câmara Técnica n° 22/2014/CTLN/COFEN, que relata: “diante da não existência de evidência científica que sustente a prática de realização da ordenha do sistema de drenagem como procedimento rotineiro para a prevenção da ocorrência de obstrução, tal prática não deve ser adotada. No entanto, em caso de obstrução do sistema, a ordenha se torna necessária”^(48)^. Uma prática bastante observada na atuação diária é a realização de pressão entre os dedos da mão em alguns segmentos do sistema de drenagem, buscando desalojar algum coágulo ou fibrina do trajeto do sistema, deslocando para o frasco coletor e possibilitando a sua remoção, a qual demonstra-se ser uma prática cientificamente validada^(29,30)^.

Dentre as limitações deste estudo, nota-se uma escassez de estudos que abordem as recomendações de cuidados aplicáveis no processo de remoção do dreno, apesar de serem condutas observadas frequentemente na prática diária. Portanto, sugere-se a realização de pesquisas mais aprofundadas sobre tais práticas, para que possam ser amplamente implementadas. Além disso, ressalta-se a falta de consenso entre os autores a despeito de algumas práticas e a escassez de estudos com alto nível de evidência que possam subsidiar tais práticas.

## CONCLUSÃO

Foram mapeados 60 cuidados de enfermagem, sendo 13 aplicáveis no momento pré-inserção do dreno de tórax, nove para durante a inserção e 38 para após a inserção do dreno de tórax. Contudo, estudos abordam ainda a falta de padronização das ações de enfermagem no cuidado ao paciente com DT e enfatizam a demanda por pesquisas robustas que possam fundamentar a implementação da PBE. Fator que pode estar associado à restrição para realização de ensaios clínicos em situações reconhecidamente deletérias aos pacientes.

As variáveis que circundam a problemática fomentam a importância do vínculo entre as instituições acadêmicas e hospitalares com o propósito da produção científica qualificada, de forma a gerar inovações e subsídios aplicáveis à assistência. Esta revisão permitiu traçar a fragilidade de evidências nos cuidados prestados a pacientes com a DT, identificando as lacunas e temas de interesse a serem abordados em novos estudos. Dentre as lacunas identificadas, pode-se citar a ausência de recomendações para o ângulo da cabeceira do leito que deve ser mantido enquanto o paciente faz uso da DT. Contudo, observa-se, rotineiramente, que pacientes com disfunções respiratórias são mantidos com a cabeceira elevada a 30° ou 45°. Além disso, destaca-se a necessidade de realização de novos estudos que possam subsidiar a prática da utilização do filtro HEPA em pacientes em isolamento por risco de aerossolização.
